# Exceptional Diversity, Non-Random Distribution, and Rapid Evolution of Retroelements in the B73 Maize Genome

**DOI:** 10.1371/journal.pgen.1000732

**Published:** 2009-11-20

**Authors:** Regina S. Baucom, James C. Estill, Cristian Chaparro, Naadira Upshaw, Ansuya Jogi, Jean-Marc Deragon, Richard P. Westerman, Phillip J. SanMiguel, Jeffrey L. Bennetzen

**Affiliations:** 1 Department of Genetics, University of Georgia, Athens, Georgia, United States of America; 2 Department of Plant Biology, University of Georgia, Athens, Georgia, United States of America; 3 Université de Perpignan, Via Domitia, CNRS UMR5096 LGDP, Perpignan, France; 4 Department of Horticulture and Landscape Architecture, Purdue University, West Lafayette, Indiana, United States of America; Fred Hutchinson Cancer Research Center, United States of America

## Abstract

Recent comprehensive sequence analysis of the maize genome now permits detailed discovery and description of all transposable elements (TEs) in this complex nuclear environment. Reiteratively optimized structural and homology criteria were used in the computer-assisted search for retroelements, TEs that transpose by reverse transcription of an RNA intermediate, with the final results verified by manual inspection. Retroelements were found to occupy the majority (>75%) of the nuclear genome in maize inbred B73. Unprecedented genetic diversity was discovered in the long terminal repeat (LTR) retrotransposon class of retroelements, with >400 families (>350 newly discovered) contributing >31,000 intact elements. The two other classes of retroelements, SINEs (four families) and LINEs (at least 30 families), were observed to contribute 1,991 and ∼35,000 copies, respectively, or a combined ∼1% of the B73 nuclear genome. With regard to fully intact elements, median copy numbers for all retroelement families in maize was 2 because >250 LTR retrotransposon families contained only one or two intact members that could be detected in the B73 draft sequence. The majority, perhaps all, of the investigated retroelement families exhibited non-random dispersal across the maize genome, with LINEs, SINEs, and many low-copy-number LTR retrotransposons exhibiting a bias for accumulation in gene-rich regions. In contrast, most (but not all) medium- and high-copy-number LTR retrotransposons were found to preferentially accumulate in gene-poor regions like pericentromeric heterochromatin, while a few high-copy-number families exhibited the opposite bias. Regions of the genome with the highest LTR retrotransposon density contained the lowest LTR retrotransposon diversity. These results indicate that the maize genome provides a great number of different niches for the survival and procreation of a great variety of retroelements that have evolved to differentially occupy and exploit this genomic diversity.

## Author Summary

Although TEs are a major component of all studied plant genomes, and are the most significant contributors to genome structure and evolution in almost all eukaryotes that have been investigated, their properties and reasons for existence are not well understood in any eukaryotic genome. In order to begin a comprehensive study of TE contributions to the structure, function, and evolution of both genes and genomes, we first identified all of the TEs in maize and then investigated whether there were non-random patterns in their dispersal. We used homology and TE structure criteria in an effort to discover all of the retroelements in the recently sequenced genome from maize inbred B73. We found that the retroelements are incredibly diverse in maize, with many hundreds of families that show different insertion and/or retention specificities across the maize chromosomes. Most of these element families are present in low copy numbers and had been missed by previous searches that relied on a high-copy-number criterion. Different element families exhibited very different biases for accumulation across the chromosomes, indicating that they can detect and utilize many different chromatin environments.

## Introduction

Transposable elements (TEs) were first discovered in maize (*Zea mays*) [1], but have subsequently been found in almost every organism investigated, from archaea and eubacteria to animals, plants, fungi and protists [2]. TEs are dynamic, abundant and diverse components of higher eukaryotic genomes, where they play key roles in the evolution of genes and genomes. The class I TEs transpose through reverse transcription of a transcribed RNA intermediate, while most class II TEs transpose through a cut-and-paste mechanism that mobilizes the DNA directly. However, there are some class II TEs, for instance *IS91* of bacteria and *Helitrons* in eukaryotes, that are believed to transpose through a rolling-circle DNA replication process that does not involve element excision [3],[4].

In most plant species, a particular type of class I element, the long terminal repeat (LTR) retrotransposons, has been observed to be the major TE, accounting for >80% of the nuclear DNA in many angiosperms [5]. The other two types of class I elements, LINEs and SINEs, have also been observed in all carefully annotated flowering plant genomes, but their copy numbers and overall contributions to genome composition have not usually been large. However, in lily (*Lilium speciosum*) and grapevine (*Vitis vinifera*), LINEs appear to be more numerous and/or active than in most plant species investigated [6],[7].

A wealth of recent studies has indicated that the class I elements, especially LTR retrotransposons, are primary contributors to the dynamics of genome structure, function and evolution in higher plants. Even within species, the LTR retrotransposon arrangement and copy number can vary dramatically in different haplotypes [8]–[11]. Some LTR retrotransposons acquire and amplify gene fragments [12],[13], and sometimes fuse their coding potential with those of other genes [14], to create “exon shuffled” candidate genes that have the potential to evolve novel genetic functions [15]. Retroelements of all types may also serve as sites for the ectopic recombination events that can cause chromosomal rearrangements: duplications, deletions, inversions and translocations. Retroelement insertions can donate their transcriptional regulatory functions to any adjacent gene, and the prevalence of this process over evolutionary time is indicated by the many fragments of retroelements and other TEs that are found in current plant gene promoters [16].

In angiosperms, polyploidy and retroelement amplification are the major factors responsible for the greater than 1000-fold variation in genome size [5]. In some lineages, amplification of only one or a few LTR retrotransposon families has been observed to more than double genome size in just a few million years [17],[18]. In other organisms, like maize, many different LTR retrotransposon families have amplified in recent times to create a large and complex genome [19].

Despite the abundance, ubiquity and genetic contributions of TEs in plants, no previous investigation has made comprehensive efforts to fully discover or characterize all of the TEs in any angiosperm genome. Even the best annotated plant genomes, those of *Arabidopsis thaliana* and rice (*Oryza sativa*), were initially examined only at a cursory level to find highly repetitive elements and those with homology to previously known TEs. Hence, subsequent studies on these genomes continue to yield new families of TEs of various types. The first exception to this rule has been the draft sequence analysis of the ∼2300 Mb maize genome, where a consortium of TE researchers has used several independent approaches in an attempt to discover and describe as many TEs as possible [20].

Even before its nearly full genome analysis, maize was the source of the best-studied TE populations in plants, including the LTR retrotransposons, where detailed analysis of small segments of the genome uncovered a great diversity of elements in different families that are mostly arranged as nested insertions [21]. The maize LTR retrotransposons were classified into 47 families [22], and comparisons between families indicated differences in their times of transposition [23], their preferential associations with different chromosomal regions [23]–[25], and their levels of expression [26].

In order to fully describe the contributions of TEs to genome structure and function, one needs to first find and describe all of the TEs in a genome. Given that that average flowering plant genome is ∼6500 Mb [27], they are expected to be composed of complex intermixtures and highly variable structures of TEs, so identification and analysis of the complete TE set will be a daunting task. Hence, we know very little about TE abundances and arrangements in anything but unusually tiny plant genomes, like those of Arabidopsis, rice and sorghum. Here, a comprehensive identification and characterization of retroelements is reported for the maize genome from inbred line B73 [20]. Hundreds of new retroelement families were discovered, and dramatic preferences in their distributions, associations and activities were uncovered. These first comprehensive studies open a window onto the true complexity of genome structure and evolution in a moderate-sized angiosperm genome.

## Results

### Retroelement discovery

In order to find all elements, LTR retrotransposons were sought by a combination of approaches that relied on both structure and homology, as described in Materials and Methods. The structure of an integrated LTR retrotransposon can be simply described as a terminal 5′ repeat that starts/ends in TC/GA), followed by a primer binding site that is used for the initiation of reverse transcription (i.e., replication), followed by polycistronic (and sometimes frame-shifted) genes that encode for several proteins necessary for element replication and integration, followed by a polypurine tract that is involved in the switch to second strand DNA synthesis, followed by the 3′ LTR. Searching for these canonical structures employed LTR_STRUC [28], combined with custom Perl scripts. All intact LTR retrotransposons were identified in a set of 16,960 sequenced maize BACs (bacterial artificial chromosomes) [20]. In addition, LTR retrotransposons homologous to known TEs in the maize LTR retrotransposon exemplar database (http://maizetedb.org/) were found by running the RepeatMasker program (vers 3.19) [29] on the assembled B73 genome using default parameters.

The element discovery process yielded 406 unambiguously distinct families of LTR retrotransposons that contained at least one intact member (Table 1), with intact being defined as the presence of two LTRs flanked by target site duplications (TSDs). Families were defined by established sequence relatedness criteria [30], and most families were named using the sequence-based criteria developed by San Miguel and coworkers [31]. Of these families, the great majority (363) were found by this structure-based screen and had not been previously described. A few (90) additional full-length LTR retrotransposons were identified that lacked sufficient structural or internal sequence information to allow one to determine their family status, and these are currently given the generic family name “unknown” (see Materials and Methods).

**Table 1:** The class I elements within the maize B73 genome.

Superfamily	# families	# new families	# homologous fragments in the B73 genome	Mb occupied in the genome	% coverage (2.045 Gb genome)	# elements containing gene fragments^1^	# families containing gene fragments
RL *Copia*	109	95	∼404,056	484.0	23.7	36	15
RL *Gypsy*	134	117	∼476,686	948.3	46.4	168	22
RL Unknown	163	151	∼221,635	92.9	4.5	221	44
SINEs	4	3	∼1,991	0.5	0.0	n.d.	n.d.
LINEs	31	13	∼35,000	20	1.0	n.d.	n.d.
Total	441	379	∼1,139,368	1545.7	75.6	425	81

1 n.d. = not determined.

LINEs were detected by their TSDs flanking a block of sequence of appropriate length (5–10 kb for L1-like superfamily member searches and 3–5 kb for RTE-like superfamily member searches), terminated on one end with a simple sequence repeat, usually poly A. Further, these candidates were required to encode at least one LINE-specific protein motif.

**Figure 1:**
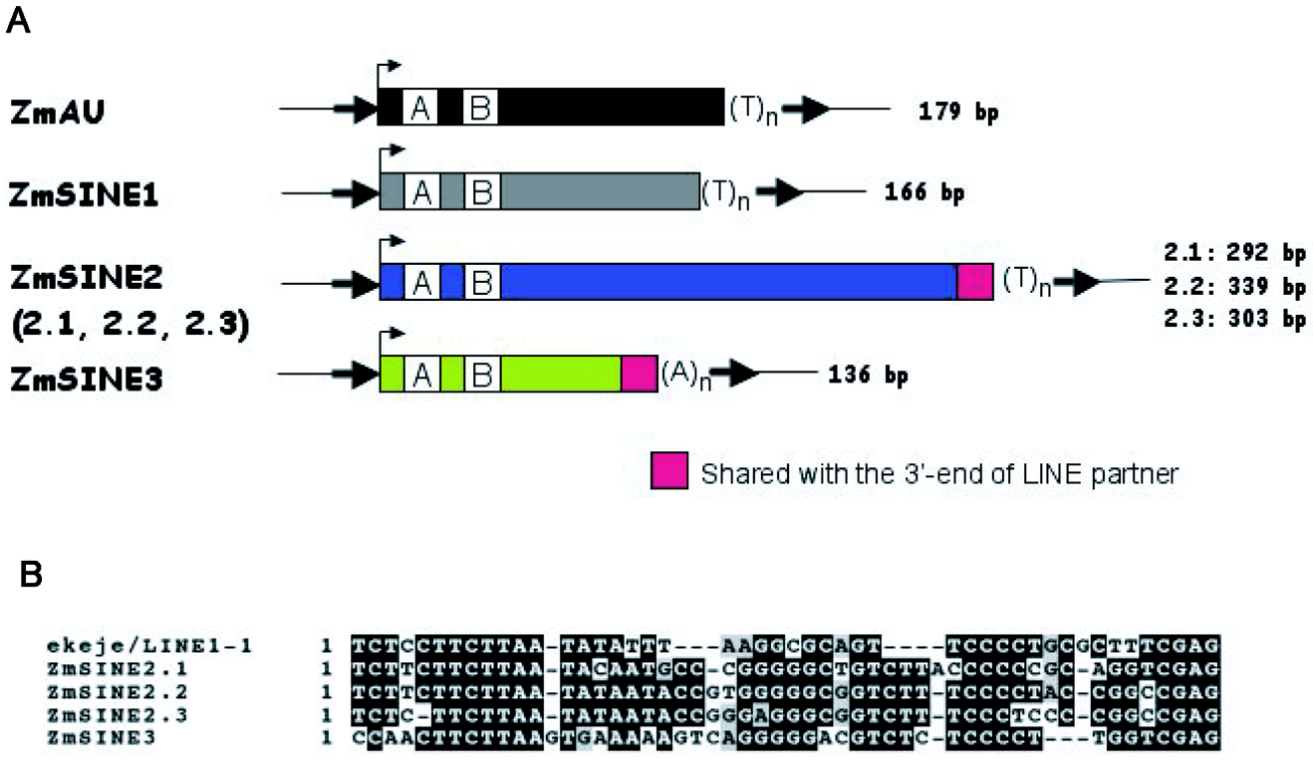
Description of the four maize SINE families. (A) Schematic representation of the four consensus maize SINEs. The size of consensus SINE sequences is indicated for each family and subfamily. The position of A and B motifs that constitute the internal (polymerase III) promoter is shown. The 3′-end similarity of ZmSINE2 and ZmSINE3 is also shown. (B) A sequence comparison of the 3′-ends of ZmSINE2.1, ZmSINE2.2, ZmSINE2.3, ZmSINE3 and the putative LINE partner, LINE1-1, is shown. No significant sequence identity (>50%) was detected between other SINE families and other maize LINE consensus sequences.

SINEs are non-autonomous retroelements that use the enzymatic machinery of autonomous LINEs to retropose (for a review see [32]). SINE discovery was mainly based on the detection of the characteristic internal RNA polymerase III promoter, as described in Materials and Methods. Prior to this search, only the ZmAU SINE family had been identified in maize [33]. Using a structure-based approach, an additional three SINE families were discovered, and are now named ZmSINE1, ZmSINE2 and ZmSINE3 (Figure 1A). All four maize SINE consensus sequences possess an internal RNA polymerase III promoter composed of conserved A and B boxes, suggesting an ancestral relationship to tRNAs. As for the pSINE family in rice and the TS SINE family in tobacco [34],[35], ZmAU, ZmSINE1 and ZmSINE2 members ends with a poly(T) stretch of 4 to more than 20 bases, a feature found only in these five plant SINE families [32]. In contrast, ZmSINE3 members end with a poly(A) stretch, a feature found for Brassicaceae SINEs [36] as well as for all other eukaryotic tRNA-related SINEs [32]. Despite this structural difference, ZmSINE2 and ZmSINE3 likely have the same LINE partner as they show strong 3′-end sequence homologies with the maize LINE1-1 consensus sequence (Figure 1B). This implies that, in the target-primed reverse transcription process leading to SINE integration by the LINE machinery, the same LINE reverse transcriptase can prime reverse transcription on a poly(A) as well as a poly(U)-ending RNA template.

### Retroelement abundance and diversity in B73 maize

Because TEs in maize and other organisms tend to insert into each other, it was possible that other TE sequences inside a retroelement might be misidentified as an intrinsic part of the retroelement. Hence, all of the retroelements identified in maize were carefully compared to the comprehensive databases for other (i.e., class I) TEs in maize [20] to produce a filtered set of retroelement sequences.

**Figure 2:**
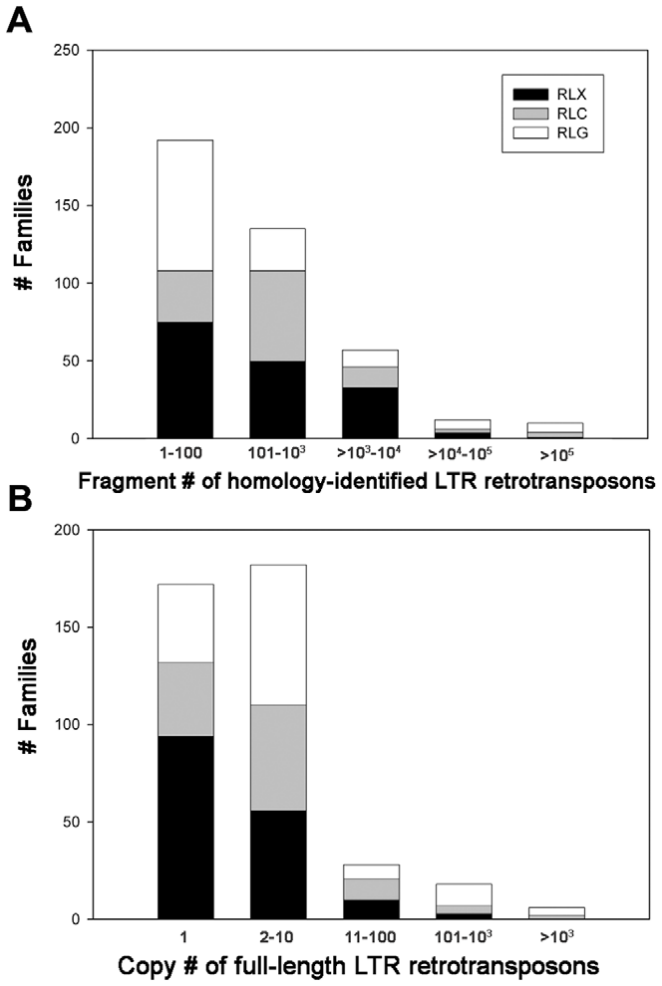
Copy number distribution of LTR retrotransposon families in the B73 maize genome. (A) The result of a homology search using the program RepeatMasker (vers. 3.19) with a library of maize LTR retrotransposon exemplars and (B) the result of a combined structure and homology screen that first uncovered the full-length LTR retrotransposons in the genome and then placed them into families, with 80% identity to an element in the exemplar database required for membership in a family.

The filtered LTR retrotransposon sequences for all 406 families were used with a RepeatMasker approach [29] to find all of the significant homologies in the B73 draft sequence [20]. At the default settings employed, similarity as small as a contiguous perfect match of 24 bp was identified as a valid homology. With this approach, over 1.1 million LTR retrotransposon fragments were identified in the B73 maize genome, contributing ∼1.5 Gb, or about 75% of the ∼2.05 Gb of the genome that has been sequenced (Table 1; [20]). As expected, the most abundant families were those that had been previously known, like *Huck*, with the four most numerous families each contributing 7–12% of the nuclear DNA. The 20 most numerous LTR retrotransposon families generate ∼70% of the sequenced B73 genome (Table 2), while the remaining 386 families mostly consist of low-copy-number families with a high diversity but lesser genomic abundance (Figure 2 and Table S1).

**Table 2:** Properties of the top 20 families that comprise ∼70% of the maize genome.

Superfamily	Family	Mb in B73, homology search	Count, homology search	Avg length, homology search	Number of FL^1^ elements, structural search	Avg length, structural search	Avg insertion date (mya), FL elements
RLG	*Huck*	233.5	59208	3943	3341	13407	1.09
RLC	*Ji*	225.8	127484	1771	4093	9523	0.77
RLG	*Cinful-zeon*	188.3	82429	2284	9844	8202	0.60
RLC	*Opie*	178.2	159512	1117	3530	8888	0.78
RLG	*Flip*	96.3	29485	3265	716	14847	0.86
RLG	*Xilon-diguus*	83.6	48297	1730	197	10964	0.77
RLG	*Prem1*	77.0	75605	1018	1479	8958	0.57
RLG	*Gyma*	64.4	39405	1635	436	12797	0.92
RLG	*Grande*	62.3	19303	3226	1338	13796	0.56
RLG	*Doke*	43.3	19523	2217	697	10630	0.74
RLC	*Giepum*	27.8	28737	968	186	12387	0.71
RLX	*Milt*	21.6	16341	1319	599	6308	1.18
RLG	*Puck*	20.7	15114	1369	514	9307	2.17
RLX	*Ruda*	19.2	42455	451	568	6485	0.74
RLG	*Tekay*	15.9	15387	1031	102	12102	0.74
RLG	*Uwum*	15.8	13271	1191	238	8495	0.80
RLG	*Dagaf*	15.8	13991	1128	185	10955	0.95
RLX	*Iwik*	8.5	18024	469	32	13874	2.29
RLC	*Wiwa*	6.8	4049	1675	162	7935	0.56
RLG	*CRM1*	6.3	3578	1761	286	6918	0.89

1 FL = Full-length elements.

Many cases were observed of gene fragments inside LTR retrotransposons (Table S2). A total of 425 intact LTR retrotransposons were observed to contain gene fragments, from a minimum of 189 independent gene fragment captures. No case was identified, under the conditions employed, where a single LTR retrotransposon contained inserted fragments from more than one standard nuclear gene. Other classes of TEs in maize are even more active in gene fragment acquisition, including 1194 gene fragment captures by *Helitrons* and 462 by other DNA transposons, including Pack-MULEs [20]. It is not known whether these gene fragments play any role in maize genetic function, for instance in the creation of a new gene or in epigenetic regulation of their donor loci.

Thirty different families (with family members defined as those with >80% sequence identity [30]) of LINEs were detected in the maize genome, with 13 of these not having been previously found and/or identified as separate families (Table 1). Approximately 35,000 LINEs (many as fragments of intact elements) were found in the B73 sequence, but this number is certain to be an overestimate caused by the many gaps and incorrect assemblies that are expected in the current maize genome draft sequence [20]. These LINEs contribute 20 Mb of DNA to the draft genome sequence, or about 1% of the total (Table 1).

Overall, SINEs represent around 0.5 Mb and 0.02% of the sequenced portion of the B73 maize genome [20]. The copy numbers are 49, 134 and 23 for the ZmAU, ZmSINE1 and ZmSINE3 families, respectively. ZmSINE2 is the major SINE family, with 1382 members. Based on phylogenetic criteria (Figure S1), the ZmSINE2 family can be further divided into three distinct subfamilies.

A phylogenetic approach was used to study the amplification dynamics of SINEs in maize. The ZmSINE1, ZmSINE2 and ZmSINE3 families contain very young members (Figure S1), close to the family consensus, suggesting very recent transposition activity. Tree topologies for these families are also typical of the “gene founder” model wherein a very small number of “master” elements are active while the vast majority of derived copies have no significant amplification potential [37]. The ZmAu family is mainly composed of more diverged members, suggesting little or no activity in the recent past.

### LTR retrotransposon superfamilies and families

In order to look at the behaviors (e.g., insertion specificities or amplification level) of the TEs across a genome, it is essential to determine their relatedness and then use this information to generate families of close relatives. Once families are generated, then family-specific behaviors can be investigated. Transposable elements of all classes tend to vary in relatedness across a spectrum, such that two TEs recently derived by transposition from the same parent element may be 100% identical in sequence, while others with a more ancient relationship can show any degree of further divergence. However, the very rapid removal of DNA from higher plant genomes [38],[39], especially from maize [40], by the progressive accumulation of small deletions indicates that TEs that last shared a common ancestor more than a few million years ago (mya) are usually largely or fully deleted from the genome. Hence, TE families can be defined by an arbitrary but consistent criterion of nucleotide sequence divergence, and a value of 80% identity has been selected by a consortium of researchers in this field [30].

In the maize genome, the classification of LTR retrotransposons into families was a major challenge because of the exceptional complexity that was observed. Nonetheless, similar to the case in the much simpler rice genome [41], all-by-all BLAST analysis of LTRs was sufficient to unambiguously define families by the 80% identity rule. Not all families could be classified in their appropriate superfamily (i.e., *copia* or *gypsy*), usually because of an absence of the genes needed for the definitive gene order criterion or for phylogenetic analysis, and these were dubbed RLX. The individual family identifications were clear, however, and each family was given a unique name. Some of these family designations conflict with previous names [42], but these earlier names were not applied with any specific rule, and thus were certain to be both misleading and temporary. For instance, the LTR retrotransposon collection called CRM [20] was actually found to represent four related, but clearly separate, LTR retrotransposon families that we have now named *CRM1*, *CRM2*, *CRM3/CentA*, and *CRM4*. Our consistent analysis using agreed-upon criteria [30] caused other such splittings of previously lumped families, and also lumped some different named families into single families that fit the 80% identity criterion (e.g., *Cinful* and *Zeon* are actually a single family that has now been named *Cinful-zeon*). The new names, and the names that had previously been applied by unspecified and/or inconsistent homology criteria, are now shown in Table S1.

### Dispersal of retroelements across the B73 maize genome

**Figure 3:**
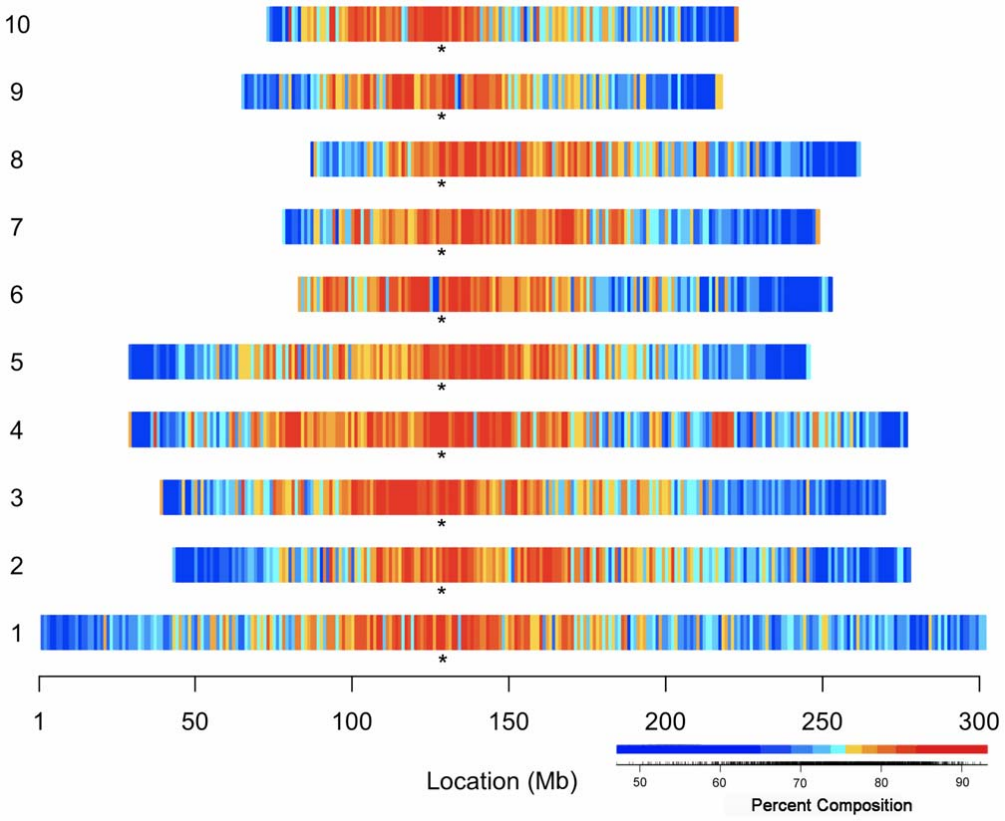
The chromosomal distribution of the LTR retrotransposon composition of the B73 maize genome. The RepeatMasker-identified LTR retrotransposons are summarized as percent composition in 1Mb bins along each of the ten chromosomes. The heatmap was derived by classifying the percent composition values into equal interval quantiles. The distribution of these classified values are illustrated as color tiles superimposed under the empirical cumulative distribution of the observed percent composition values. Asterisks indicate approximate centromere positions.

The assembled physical and genetic map of maize inbred B73 [20] allows placement of any class of sequence along that portion of the genome that was sequenced. Overall, LTR retrotransposons are found to be most abundant in pericentromeric heterochromatin and least abundant in the more gene-rich arms on all chromosomes (Figure 3). However, different LTR retrotransposons are found to be differentially clustered in such analyses, with the general observation that the *gypsy* superfamily of LTR retrotransposons is concentrated in the pericentromeric heterochromatin while the *copia* superfamily shows a preferential accumulation in the more euchromatic regions of the chromosome arms [20]. Despite this general pattern, individual families show deviations from the rule. For instance, the *gypsy* family *Huck* was found to exhibit a more ‘*copia*-like’ distribution on chromosome 1 (Figure S2). Another *gypsy* family, *Grande*, shows a relatively even distribution across 10 Mb bins of this same chromosome. Hence, there are families that accumulate in a pattern that contrasts with the general behavior of their superfamilies in maize.

**Figure 4:**
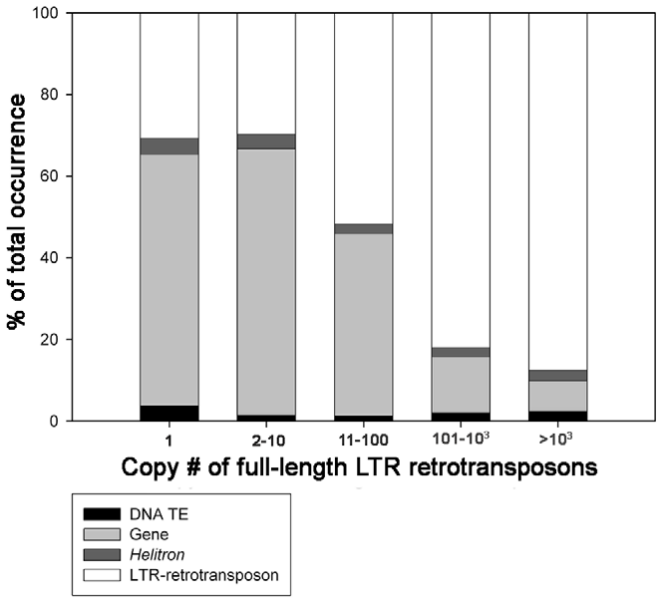
The insertion-site preferences of maize LTR retrotransposons. The full-length LTR retrotransposons were placed into bins according to their relative copy number and the results of blast analysis to separate databases of maize genes, cut-and-paste DNA TEs, *Helitrons*, and LTR retrotransposons were summarized according to their copy number classes.

A more dramatic correlation between LTR retrotransposon family property and insertion/accumulation pattern was observed by comparing the copy numbers of intact elements in a LTR retrotransposon family with the nature of the sequences within 500 bp (on each side) of the insertion site. Low-copy-number families were found to be most often inserted into the regions in or near genes (or gene fragments), while high-copy-number families were observed to primarily accumulate inside other LTR retrotransposons (Figure 4).

LINEs of both RIT and RIL (L1-like) families were found to be fairly evenly distributed across all chromosomes, with a higher abundance in distal regions of the chromosomes (Figure S3). Although maize LINEs have been observed to show a preferential association with genic regions, especially introns [43], their common occurrence in pericentromeric DNA suggests that many insertions are not in or near genes.

Of the 1991 SINEs discovered, 1174 were found in the introns or UTRs (untranslated regions) of genes and 21 in putative coding exons (data not shown). Only 796 were found in the intergenic space that makes up more than 85% of the sequenced B73 genome [20]. Hence, like SINEs in other species, these small TEs show a very strong preference for association with genes in the maize nuclear genome. In this regard, the general distribution of SINEs across the maize chromosomes (Figure S4) was found to exhibit a pattern quite similar to the gene distribution [20].

### Correlated patterns of retroelement distribution

As previously observed in other organisms by numerous scientists studying many different genomes, maize TEs were found to make up a greater quantity of the total DNA in the gene-poor pericentromeric regions than in other parts of the genome (Figure 3). However, as mentioned above and observed previously (reviewed in [44]), LINEs, SINEs and some LTR retrotransposon families accumulate preferentially in areas that are near genes.

**Figure 5:**
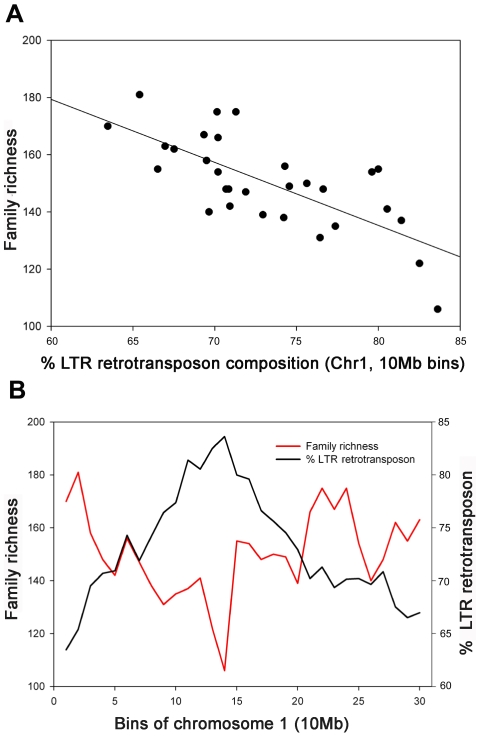
Abundance and family richness of LTR retrotransposons found on chromosome 1. (A) The relationship between the % LTR retrotransposon abundance and family richness per 10 Mb bins, and (B) the specific pattern of abundance and richness plotted along the chromosome.

Figure 5 shows the relationship between LTR retrotransposon abundance and LTR retrotransposon family richness across chromosome 1 of maize inbred B73, and this general pattern was found to be the same across all other chromosomes (data not shown; Table S3). Hence, on all maize chromosomes, those regions that have the most total LTR retrotransposons also have the fewest kinds of LTR retrotransposons. This observation echoes the relationship between the number of species and the abundance of individual species in both terrestrial and aquatic environments, but has no precedent that we are aware of in TE studies.

**Figure 6:**
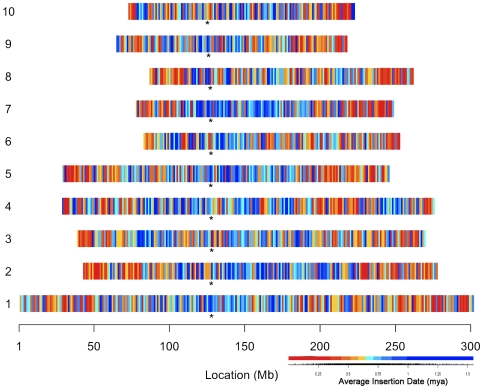
The chromosomal distribution of full-length LTR retrotransposon insertion histories. The insertion date of each full-length LTR retrotransposon was determined and these values were averaged for all full-length LTR retrotransposons occurring in each 1 Mb bin. The heat map was derived by classifying the average insertion age into equal-interval quantiles. The distribution of these classified ages are illustrated as color tiles superimposed under the empirical cumulative distribution of the average insertion dates for each bin. Asterisks indicate approximate centromere positions.

The insertion dates of intact LTR retrotransposons was observed to vary according to the distance from the centromere. Younger elements are enriched in the euchromatic regions whereas older elements are most abundant in the pericentromeric regions (Figure 6). An analysis of variance showed that the average insertion date per 1 Mb bin varied according to distance from the centromere (F = 2.08; P<0.0001), and this relationship held across most of the chromosomes (Table 3).

**Table 3:** An analysis of variance showing the relationship between LTR retrotransposon insertion date and the distance to the centromere.

Chromosome	df	Type III SS (10^5^)	F-value	P-value
1	165	41.45	1.41	0.020
2	143	37.50	0.8	0.886
3	134	30.44	1.43	0.033
4	140	41.01	1.33	0.062
5	107	32.07	1.4	0.044
6	118	28.90	1.36	0.110
7	114	37.99	1.07	0.399
8	126	24.07	0.69	0.945
9	82	23.82	1.74	0.010
10	88	35.71	2.1	0.001

Distance to the centromere in 1 Mb bins was the dependent variable whereas the square-root transformed average insertion date per 1 Mb bin was the independent variable.

**Figure 7:**
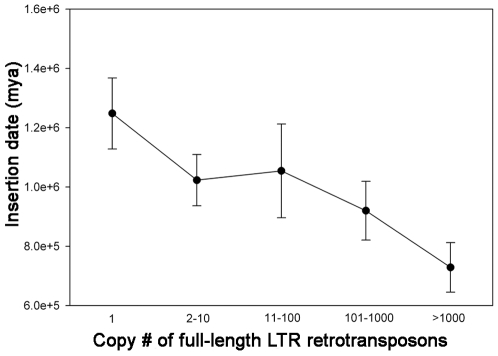
The average date of LTR retrotransposon insertion for each of the copy-number classes.

The average date of LTR retrotransposon insertion for a given family was also observed to correlate with the current perceived copy numbers of the LTR retrotransposon families. As a general pattern, the lower-copy-number elements were more ancient insertions (averaging about 1.2 mya) compared to the highest-copy-number elements (averaging about 0.7 mya) (Figure 7). Because most of the higher-copy-number LTR retrotransposons are of the *gypsy* superfamily (Table 2), and show an overall pericentromeric accumulation bias [20], one expected the opposite result because of the slower rate of LTR retrotransposon removal in gene-poor (and thus recombination-poor) regions like the pericentromeres [45].

## Discussion

### Limitations of the dataset and problems this might generate

The landmark sequencing of the very complex and fairly large maize genome was accomplished at a small fraction of the cost of previous clone-by-clone sequencing projects because of the expertise of the researchers involved, a low redundancy of initial shotgun sequencing, and because of a decision to not finish any regions of the genome that appeared to lack gene candidates [20]. Hence, a very comprehensive TE discovery and masking process was necessary to facilitate finishing that was efficiently targeted on genes. One disadvantage of this approach, however, is that most sequenced regions are composed of many tiny contiguous sequences (contigs). Our analysis of the current B73 assemblies (data not shown) indicates a median contig size of ∼7 kb with ∼60% of the assembly occurring in contigs larger then 30 kb. Thus, a structure-based search approach that requires intact elements, like the one employed in this project, will miss any families where the only intact members are fractured by sequence gaps or inaccurate scaffolding of contigs. This is expected to be most problematic for large TEs (like LTR retrotransposons) and for those that only have a few intact members. Hence, our prediction that ∼75% of the B73 maize genome is composed of LTR retrotransposons is a minimum estimate.

Also because of the many tiny sequence gaps in the assembly, there will be many occasions when an intact retroelement was identified by RepeatMasking as several fragments of an element. Hence, calculation of the ratio of intact to fragmented LTR retrotransposons is not valid with this dataset. In contrast, this same analysis with the random sampling of fully sequenced and annotated clones known as the GeneTrek approach does allow accurate quantification of the relative abundance of different TE structures. In such a GeneTrek analysis, the ratio of intact to truncated LTR retrotransposons in maize was found to be ∼2∶1 [40],[46], quite different from the ratio of ∼1∶27 that was calculated (Baucom and Bennetzen, data not shown) as an artifact of this same analysis on the currently fractured B73 assembly [20].

There are also many large sequence gaps, and numerous sequenced BACs with no home in the assembly, for the B73 draft sequence [20]. It is likely that about 90% of the maize nuclear genome is present in the current assembly (∼2005 Mb out of ∼2300 Mb). From all previous full genome sequences in multicellular eukaryotes that have centromeres, the standard observation has been that the majority of the unsequenced regions are in the gene-poor areas around the centromeres and in other heterochromatic blocks. Because these gene-poor chromosome segments also tend to be LTR retrotransposon-rich, these results provide a further reason to believe that the B73 maize genome contains more than 75% LTR retrotransposons, with an upper limit of ∼85%.

Importantly, however, the overall quantitation of retroelement contributions to the B73 genome is not dramatically biased by the gaps and other intrinsic errors in the current assembly. As shown in Figure S5, most LTR retrotransposons exhibit the same relative abundance when used to mask the current B73 draft assembly as they do when used to mask a shotgun dataset from the same B73 line (R^2^ = 0.99, p<0.0001). The few exceptions to this observation (e.g., *Ipiki*) are likely to be LTR retrotransposons that are preferentially abundant in that ∼10% (e.g., near centromeres?) of the maize genome that is not present in the assembly [20].

Previous maize studies had uncovered primarily the high-copy-number retroelements [21],[23], with some exceptions of low-copy-number TE discovery associated with particular mutations [47],[48] or carefully sequenced and annotated small segments of the maize genome [46]. All of the LTR retrotransposons found in these earlier studies were also found in this analysis, at the approximate predicted frequencies. The major difference, however, was the large dataset available in the current study, and thus the discovery of hundreds of additional LTR retrotransposon families. Only by this comprehensive analysis on the majority of the maize genome was it possible to determine the exceptional complexity of retroelements in maize, and their different properties of dispersal and divergence.

### Diversity and its meaning

Rice, with an ∼400 Mb nuclear genome, has 172 identified LTR retrotransposon families that contribute ∼97 Mb, distributed across 48% with only a single intact element, 20% with 2 intact elements and 32% with 3 or more intact elements [41]. Maize, in contrast, has 406 identified LTR retrotransposon families, just over twice as many, but they contribute ∼1700 Mb of DNA to the maize nuclear genome. These maize elements are distributed across 42% singleton intact elements, 21% with 2 intact elements and 37% of families with 3 or more intact elements. Hence, the >17X greater amount of LTR retrotransposons in maize compared to rice is not primarily caused by a greater number of element families in maize but instead by a much higher copy number of a very small number of superabundant families.

Two of the many misconceptions about TE properties in higher eukaryotes are that they are highly repetitive and are randomly scattered about the genome. In fact, many TE families are present in very low copy numbers. The median family copy number of intact LTR retrotransposon with TSDs in B73 maize was measured to be 2 (mean ∼77), with a total of 256 families that contained only one or two intact LTR retrotransposons that were detected. Most LTR retrotransposon families are distributed quite unevenly across the genome, probably an outcome of both differences in insertion preferences and different rates of LTR retrotransposon removal in different chromosomal locations [44]–[46],[49]. The previous observation that LTR retrotransposons show a dramatic bias in whether they insert into LTRs or the internal regions of other LTR retrotransposons [21] was not observed, however, and it now seems likely that the previous conclusion was an artifact of a small sample size.

Studies in rice and other organisms suggest that LTR retrotransposons are more rapidly removed (sometimes by unequal homologous recombination to generate solo LTRs) in regions with high recombination rates, like areas around genes and in the cores of centromeres [45],[46]. One example of this analysis was that the ratio of solo LTRs to intact elements was found to be much higher in gene-rich and recombination-rich euchromatic regions than in gene-poor and recombination-poor pericentromeric regions [44]. Although natural selection should also more rapidly remove individuals from a population that contain retroelements or other TEs detrimentally inserted into coding and gene regulatory regions, this process alone cannot explain the differential retroelement accumulation properties that we observe. For instance, why would LINEs, SINEs and low-copy-number LTR retrotransposons not be depleted in genic regions, while high-copy-number LTR retrotransposons are? A simpler explanation is that different retroelements are directed to preferentially insert in different parts of the genome by the biases of their integrases for association with specific chromatin proteins, as observed with Ty elements in yeast [50].

We have no idea how many types of DNA::protein configurations are actually present in plants, of course, but it is very clear that chromatin consists of more than just hetero- and eu- varieties [51], so sufficient variability should be present to allow a great wealth of different TE insertion specificities, as has been recently reported in *Arabidopsis* [52]. Particularly fascinating are the high-copy-number LTR retrotransposons like *Ji* and *Opie* that preferentially avoid insertion into genes, but primarily insert into heterochromatin near genes, while other high-copy-number elements like *Gyma* avoid inserting into genes or heterochromatin near genes, preferring instead an accumulation into large gene-free heterochromatic blocks [46]. Unlike low-copy-number LTR retrotransposons, which are associated with *de novo* mutations in many plant species, neither class of high-copy-number LTR retrotransposons is associated with a mutation caused by insertion into a gene. Perhaps TE insertion profiles will be a uniquely useful route to uncover and map a broad spectrum of novel chromatin structures.

### Retroelement distribution and the origin of plant-genome complexity

Genomic complexity is not just a matter of the number of different sequences, but also of the variability in their arrangement and stability. The factors that determine differences in these arrangements, such as differential insertion specificities and differences in retention, are only beginning to be understood. It is already clear, though, that TE insertion and retention biases are the major forces that determine local genome structure in maize and other complex plant genomes. The mechanisms responsible for these biases, and their outcome vis-à-vis gene/genome function and evolution, are only now beginning to be understood.

Viewed from the standpoint of the TE, much of the diversity in TE populations and their arrangement takes on a new and informative light. A previous model proposed that low-copy-number TEs must insert near or into genes so that they have a reasonable chance of expression and activity in subsequent generations, while highly repetitive TEs need to avoid insertions that disrupt genes in most cases because 1000 or 10,000 such insertions would lead to a dead host [44]. Hence, abundant TEs rely on their abundance *per se* to guarantee transmission and the opportunity for activity in future generations. The data for LTR retrotransposon abundance versus copy number shown here agrees with this model, as does the fact that (to date) none of the high-copy-number LTR retrotransposons have been shown to cause a *de novo* mutation, while low-copy-number LTR retrotransposons (e.g., *Bs1*, *Tnt1*, *Tos17*) that make up a relatively small part of their genomes have caused many new mutations [47]–[49],[53]. The analysis of the maize genome suggests that the copy number for this transition is fairly low, 10–100 intact copies per genome (Figure 4), for this change in lifestyle. LTR retrotransposon families with copy numbers less than ten were usually found to preferentially accumulate in genic regions, while most LTR retrotransposon families with copy numbers higher than 100 were found to be enriched in gene-poor regions like pericentromeric heterochromatin.

The insertion preferences of LTR retrotransposons can contribute to their potential for more than just transcriptional activity. Elements that land in recombination-rich regions have a greater chance of inter-element unequal events that can create novel LTR retrotransposons with possible new properties [38]. Insertion into an LTR provides the opportunity to acquire the gene regulatory properties of the target LTR retrotransposon. Moreover, insertion of an LTR retrotransposon into an LTR retrotransposon would usually eliminate the target element as a potential competitor for future amplification.

The observed relationship between LTR retrotransposon family richness and LTR retrotransposon abundance across the maize chromosomes is the most compelling indicator, in this study, of the validity of the conceptualization of TEs as competitor organisms whose world is the nuclear genome. When an environment is highly suitable for proliferation of a category of life, a few highly adapted types of individuals (e.g., species or, in this case, LTR retrotransposons) crowd out all other competitors to create a dense but diversity-poor ecosystem. Other species, here proposed to be the lower-copy-number LTR retrotransposons, disseminate themselves at lower abundances across less productive environments that thus become diversity-rich. Of course, it is not at all clear what aspect(s) of these TE-enriched regions might make them “productive” from a TE perspective. Perhaps it is something as simple as a lower rate of TE removal by ectopic recombination [45]. This view of genomic life provides another angle to investigate TEs, as highly adapted commensals, but in no way suggests that they cannot be utilized when the opportunity arises for a process that benefits the plant host. The occasional creation of new genes by TE capture and shuffling of gene fragments or through fusion of TE genes (or regulatory regions) with nearby genes falls into this category. What remains constant in these considerations is the long-term evolutionary value of the instability and diversity generated by retroelements and other TEs.

## Materials and Methods

### Generation of the maize LTR retrotransposon exemplar database

New families of maize LTR retrotransposons were discovered by several iterations of masking and re-investigation. First, 5,075 maize BACs were downloaded on February 22, 2007 from the Washington University maize sequencing project [20] and masked using the RepeatMasker program [29] with a database of previously known maize LTR retrotransposons. Masked regions were removed from the sequence, and LTR_STRUC [28] was used to find new elements. This program identifies LTR retrotransposons based on the presence of LTRs, matching target site duplications (TSDs), and the presence of the canonical TG/CA motif found at the 5′ and 3′ end of each LTR (although deviations are permitted), and thus is a structure-based screen rather than one that requires sequence homology to a known TE. This process was designed to uncover old and fragmented families of LTR retrotransposons after masking out the younger and previously discovered families [21],[22].

Next, 15,708 maize BAC sequence data sets were downloaded March 1, 2008 from the Washington University sequencing project and were first masked at a quality score of ‘40,’ then screened with LTR_STUC. 13,362 LTR retrotransposons were found and, along with the sequences uncovered in the initial screen, placed into families using the RepMiner classification tools (http://repminer.sourceforge.net/) [54]. This process generated ∼600 maize LTR retrotransposon exemplar sequences that best describe each of 412 identified families. Each exemplar was annotated for LTR position, the primer-binding site sequence and the genes involved in the transposition process.

Exemplars were identified as members of either the *copia* or *gypsy* superfamilies based on the position of the reverse transcriptase gene in relation to the integrase gene, and by using a maximum-likelihood gene tree of reverse transcriptase. Both methods of superfamily designation were 100% congruent. Exemplar sequences that did not contain internal coding regions with an identifiable homology to LTR retrotransposon genes were given the ‘unknown’ superfamily designation. Each exemplar was hand-curated to ensure that exemplars where not chimeric annotations that contained insertions of other LTR retrotransposon sequences. DNA transposons inserted within the LTR retrotransposon exemplars were identified by homology-based searches against the maize TE database (http://maizetedb.org/) and were excluded from the exemplar sequence by masking.

Family nomenclature follows established methodology [30] in which the TE classification can be deduced from the full family name. In this system, family names are given a three character prefix that represents the class, order and superfamily of the individual family. For example, families with the RLG prefix represent LTR retrotransposons that are members of the *gypsy* superfamily while the RLC prefix represents families that are members of the *copia* superfamily. LTR retrotransposons that could not be assigned to the *gypsy* or *copia* superfamilies were assigned the RLX prefix.

### Annotation of LTR retrotransposon distribution with RepeatMasker

The B73 maize genome represented as an Accessioned Golden Path (AGP) assembly [20] was downloaded from the Arizona Genomics Institute (http://www2.genome.arizona.edu/genomes/maize). This dataset was investigated for LTR retrotransposon content using the default settings in RepeatMasker [29] with the curated exemplar library of maize LTR retrotransposons (http://maizetedb.org/).

The RepeatMasker annotation of the maize AGP assembly was uploaded to a custom MySQL relational database to facilitate manipulation and querying of sequence features mapped onto the maize genome assembly. The RepeatMasker output files derived from masking the AGP with the exemplar database were translated to General Feature Format (GFF) style coordinates using the cnv_repmask2gff.pl program [55]. These coordinates were uploaded to a MySQL database using custom Perl scripts. The database served as the query engine to trim overlapping features resulting from the RepeatMasker annotation and provided the framework to query distribution related information. The MySQL database schema and custom Perl scripts used to generate the non-redundant distribution information are available from the authors upon request.

Each of the AGP chromosomes was spatially binned into 10 Mb non-overlapping units and the percent LTR retrotransposon composition within each bin was determined, as was the number of distinct families present within each bin. The strength and direction of the correlation between percent LTR retrotransposon composition and family richness was determined using the Resample program [56] separately for each chromosome.

### Identification, classification, and location of full-length LTR retrotransposons

The sequence files for the 16,007 BAC assemblies incorporated in the maize AGP were downloaded from GenBank. Full-length LTR retrotransposons were identified by LTR_STRUC and mapped onto these BACs through the use of batch annotation scripts available in the DAWGPAWS annotation package [55]. This process resulted in a database of 35,229 full-length LTR retrotransposons.

The 5′ LTR sequences of this dataset of full-length LTR retrotransposons were used to classify the elements into families using at least 80% identity in a BLASTn analysis employing the exemplar database. LTR retrotransposons that were not homologous to families present within the exemplar database (1,979) were removed from analysis, with the exception of the gene capture analysis, explained below. Further, sequences that were 2 standard deviations greater in length than the assigned family's mean length (2,135) were also removed from analysis. These sequences were found to harbor full-length insertions of other LTR retrotransposons and thus do not provide an accurate characterization of the most recently intact elements. The resultant database of full-length LTR retrotransposons consisted of 31,115 individual sequences distributed among 406 distinct families. Six families initially identified on the maize BACs used to create the exemplar database were not found in the current assembly of the AGP, potentially due to the fact that 981 BAC sequences released from the Washington University sequencing effort were not used to assemble the AGP. The location of full-length LTR retrotransposons on the AGP was determined using the data conversion table provided by the Arizona Genomics Institute.

### LTR retrotransposon insertion history and specificity

The insertion date of each full-length LTR retrotransposon was determined by estimating the amount of divergence between the 5′ and 3′ LTRs [23]. Perl programs were used to automate this process; the two LTRs of each mined LTR retrotransposon were first aligned using ClustalW [57], and the genetic divergence between the two was estimated using the baseml module of PAML ([58], vers. 4). The time since insertion of each LTR retrotransposon element was estimated using the substitution rate of 1.3×10^−8^ per site per year [11]. To determine if distance to the centromere explained variation in insertion dates, the GLM procedure of the SAS statistical package (vers. 9.2) was used to perform an analysis of variance with the square-root transformed average insertion date per bin as the dependent variable and the distance of each bin to the centromere as the independent variable. This analysis was performed separately for each chromosome.

Investigation into the insertion-site specificity of each full-length LTR retrotransposon was conducted by a performing a BLASTn search to four separate databases, namely those containing maize genes [20] and those containing DNA transposable elements, *Helitrons*, and LTR retrotransposons (http://maizetedb.org/). 500 bp of maize sequence flanking the 3′ and 5′ sides of each element was used as the query in separate nucleotide BLAST analyses, and the results were parsed for at least 80% identity. No annotations >5 bp away from the query sequence were included, because the objective was to determine what type of sequence the LTR retrotransposons inserted into, rather than those sequences that were simply nearby.

### LTR retrotransposon capture of host gene fragments

A set of curated genes from the rice genome (RAPDB, vers. 4) was used to search the full-length maize LTR retrotransposons for instances of host gene capture. The full-length LTR retrotransposon dataset was screened for homology to rice genes at an Expect value of e^−5^. Significant BLAST hits were screened for TE genes, and genes were also removed if annotated as ‘rice gene family candidate’ and present in high copy number (>20), as they are likely to be undiscovered TE genes. The full-length LTR retrotransposons that were not placed into families based on the 80% identity rule were retained in this analysis as they represented ∼20% of the total gene capture events. The annotations of these particular LTR retrotransposons indicated that they exhibit general LTR retrotransposon features, such as target site duplications and a TG/CA motif at the end of the LTRs, and as such represent LTR retrotransposons of ‘unknown’ family classification.

### Maize shotgun data

Trace files of whole genome shotgun (WGS) DNA sequence reads for maize inbred B73 were obtained from those deposited by the Joint Genome Institute (JGI) to the NCBI Trace Archive (http://www.ncbi.nlm.nih.gov/Traces/trace.cgi?). These sequence files were trimmed of low quality bases and vector sequence using Lucy [59]. Organellar sequences were identified by BLAT [60]. Alignments to maize chloroplast and mitochondrial DNA and were removed from further analysis. This filtering resulted in a dataset of 1,028,203 high quality sequence reads totaling 79,6326,632 bp of genomic DNA. These data represent an approximately one-third sample sequence coverage of the B73 genome.

The JGI shotgun data were annotated for LTR retrotransposons using RepeatMasker ([29], vers. 3.19) with the same database and parameter set used to annotate the AGP. Overlapping features from the RepeatMasker output were identified using the same methodology described for LTR retrotransposon annotation of the maize AGP assembly. Significant outliers between the ratios found in the AGP and the ratios found in the JGI shotgun data were identified by performing an outlier analysis in the SAS statistical package (vers. 9.2).

### SINE detection

The approach to identify potential SINE families was divided into several steps. The first step was the search for anchors, which were defined as small regions containing SINE features (see below). Following that, a 500 bp region flanking the anchor on each side was extracted. These sequences were used to perform a non-stringent search for direct repeats (likely to be TSDs) that were less than 350 bp apart. The sequences that passed the filter were aligned using ClustalW [57], alignments were refined using muscle [61] and corrected by hand using Seaview [60].

A first approach for SINE identification consisted in developing an hmm model using hmmer (http://hmmer.wustl.edu) for the region harboring the main anchor, which is the internal (tRNA-related) promoter for RNA polymerase III, defined for SINEs as an “A” box (RRYNNRRYGG) around position +14 of the start of the repeat and a B box (GGTTCGANNCC) around position +54 of the start of the repeat. This anchor was designed using known plant SINE elements. This model was then used to search the whole pseudomolecule representing the draft sequence of the B73 maize genome [20]. A second approach consisted in identifying tRNAs using tRNAscan-SE and using those sites described as “Pseudo tRNAs” as anchors. A third approach consisted in using the last 30 bases of maize LINE consensus sequences to screen for homology by BLASTn against the B73 draft genome, and to then use these homologies as anchors. In this case, to make sure that SINEs were distinguished from severely truncated LINEs, these homologies were searched for the presence of internal A and B boxes typical of tRNA-derived SINEs. A search for 5S RNA-derived SINEs was also performed, using as anchor the A/IE/C conserved boxes of the 5S RNA internal polymerase III promoter, without success. SINEs that did not share significant sequence identity (<50%) outside of the common SINE features (internal polymerase III promoter and 3′-terminal end) were classified in distinct families. For SINEs that do have significant homologies (>50%) outside of the common SINE features (>50%), further subfamily classifications were proposed using phylogenetic criteria.

### SINE phylogenetic analysis

The SINE sequences were aligned using the ClustalW multiple-alignment program [57] with some manual refinements (i.e., elimination of unnecessary gaps at the beginning and end of the ClustalW alignment). Evolutionary distances were calculated using the Jin-Nei distance method of the Dnadist program (PHYLIP package version 3.573c [62]. The coefficient of variation of the Gamma distribution (to incorporate rate heterogeneity) and the expected transition to transversion ratio (t) were obtained by pre-analyzing the data with the Tree-Puzzle program [63]. Phylogenetic trees were inferred using the Neighbor-Joining (NJ) method (PHYLIP package version 3.573c [62]). Consensus trees were inferred using the Consense program (PHYLIP package). The significance of the various phylogenetic lineages was assessed by bootstrap analyses [64].

## Supporting Information

Figure S1 Comparison of maize SINE evolution histories. (A) ZmAU, (B) ZmSINE1, (C) ZmSINE2, and (D) ZmSINE3. All full-length or near full-length elements were analyzed. The phylogenies were obtained using the Neighbor-Joining method. Significant bootstrap values are shown. The nucleotide divergence scale is indicated for each phylogeny.
(9.24 MB TIF)

Figure S2 The distribution of LTR retrotransposon family abundance across chromosome 1.
(1.12 MB TIF)

Figure S3 Distribution of LINEs across chromosome 1.
(8.01 MB TIF)

Figure S4 The general distribution of SINEs across the maize chromosomes. Different colors indicate different SINE families, as indicated in the figure.
(0.16 MB TIF)

Figure S5 The relationship between the abundance of LTR retrotransposon families found within the AGP compared to their abundance in the sample sequence. Significant outliers are noted on the figure.
(0.31 MB TIF)

Table S1 Properties of all maize LTR-retrotransposon families examined in this manuscript.
(0.07 MB XLS)

Table S2 Gene capture events uncovered in the full-length LTR-retrotransposons.
(0.03 MB XLS)

Table S3 The re-sampled correlation coefficients describing the relationship between % LTR retrotransposon.
(0.01 MB XLS)
