# Supplementary figures and images for: Exceptional Diversity, Non-Random Distribution, and Rapid Evolution of Retroelements in the B73 Maize Genome
Source: PLoS Genet. 2009 Nov 20;5(11):e1000732. doi: 10.1371/journal.pgen.1000732 (PMC2774510; doi:10.1371/journal.pgen.1000732)

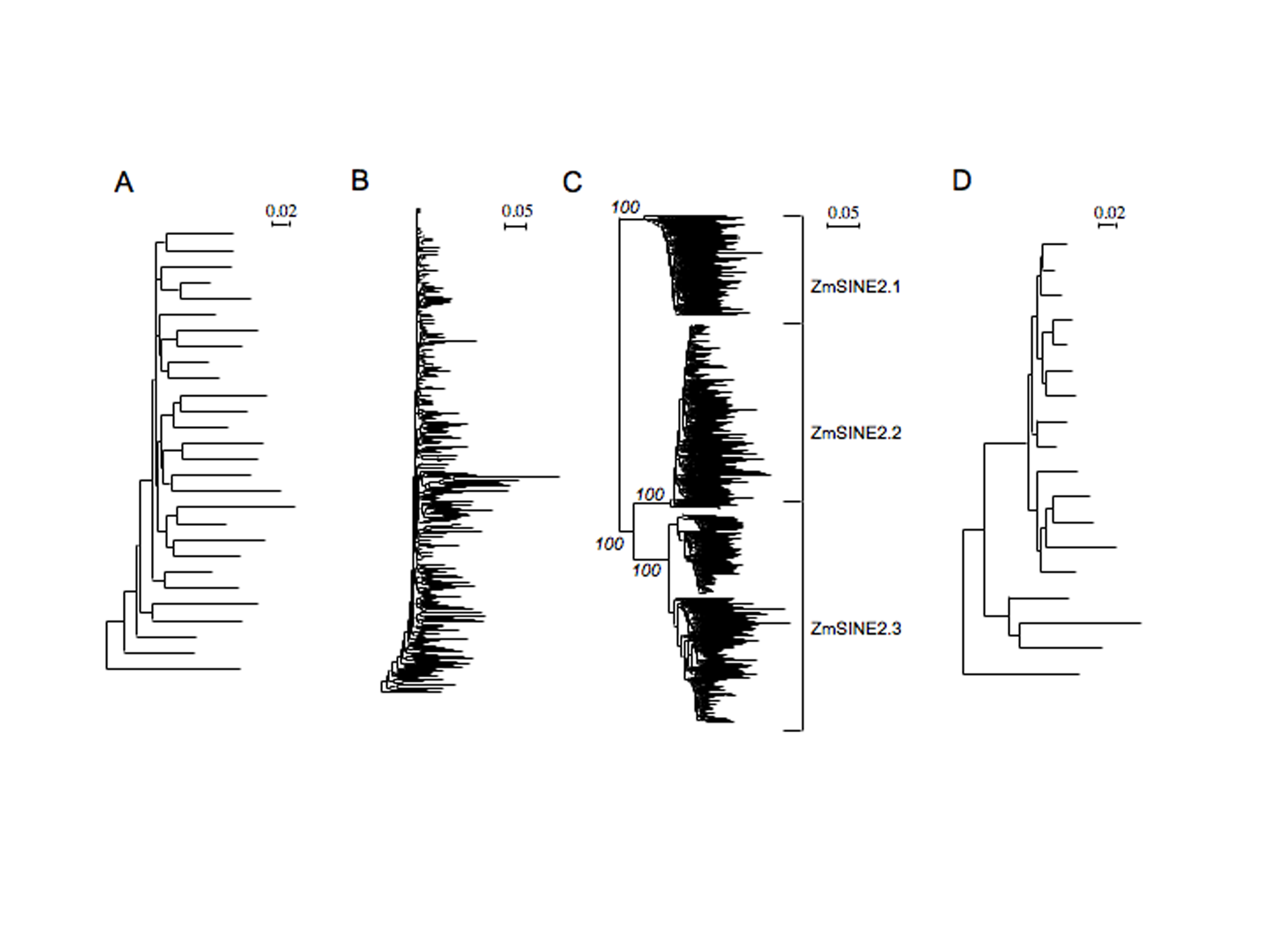

Supplement: Figure S1 — Comparison of maize SINE evolution histories. (A) ZmAU, (B) ZmSINE1, (C) ZmSINE2, and (D) ZmSINE3. All full-length or near full-length elements were analyzed. The phylogenies were obtained using the Neighbor-Joining method. Significant bootstrap values are shown. The nucleotide divergence scale is indicated for each phylogeny. (9.24 MB TIF) [file pgen.1000732.s001.tif]

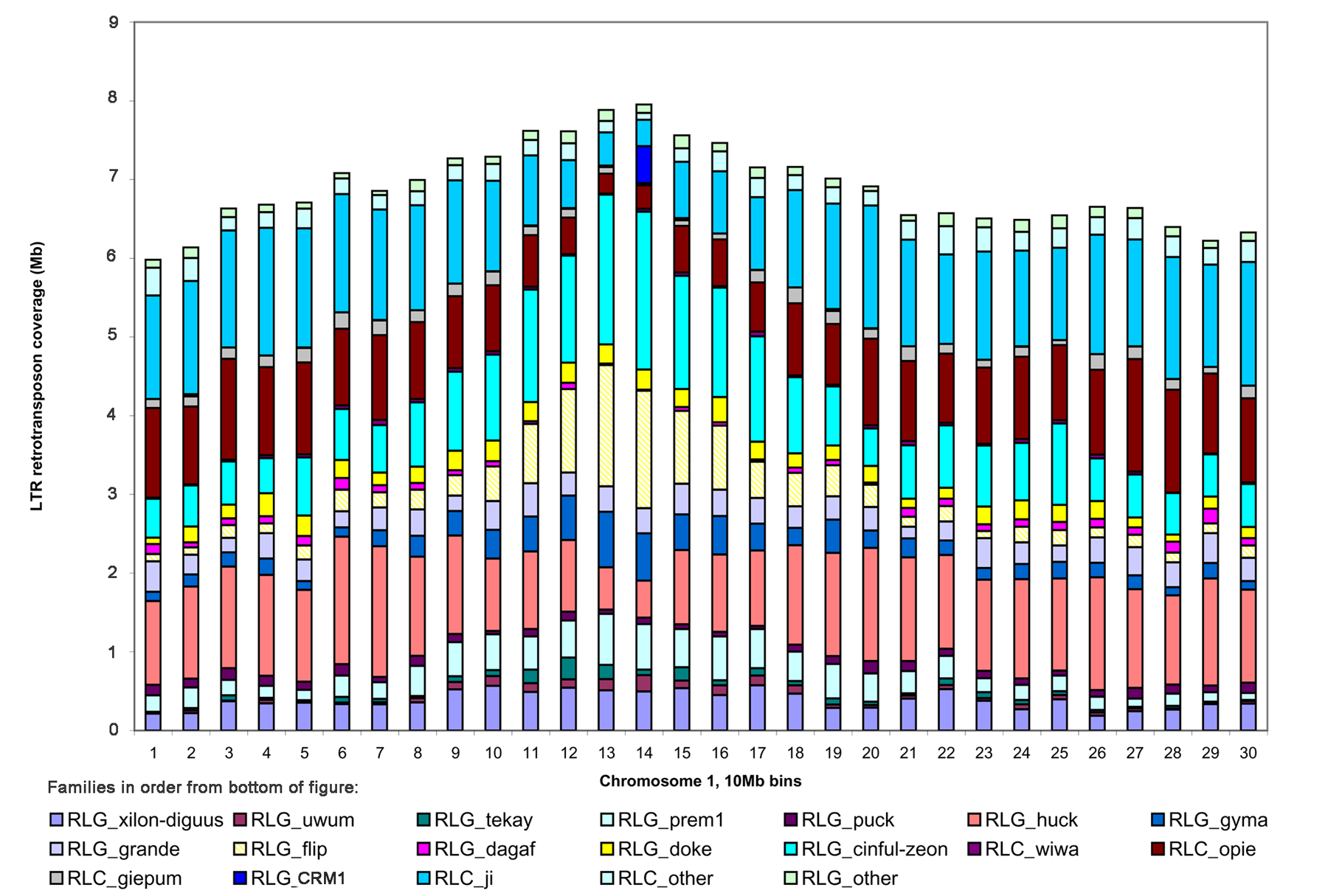

Supplement: Figure S2 — The distribution of LTR retrotransposon family abundance across chromosome 1. (1.12 MB TIF) [file pgen.1000732.s002.tif]

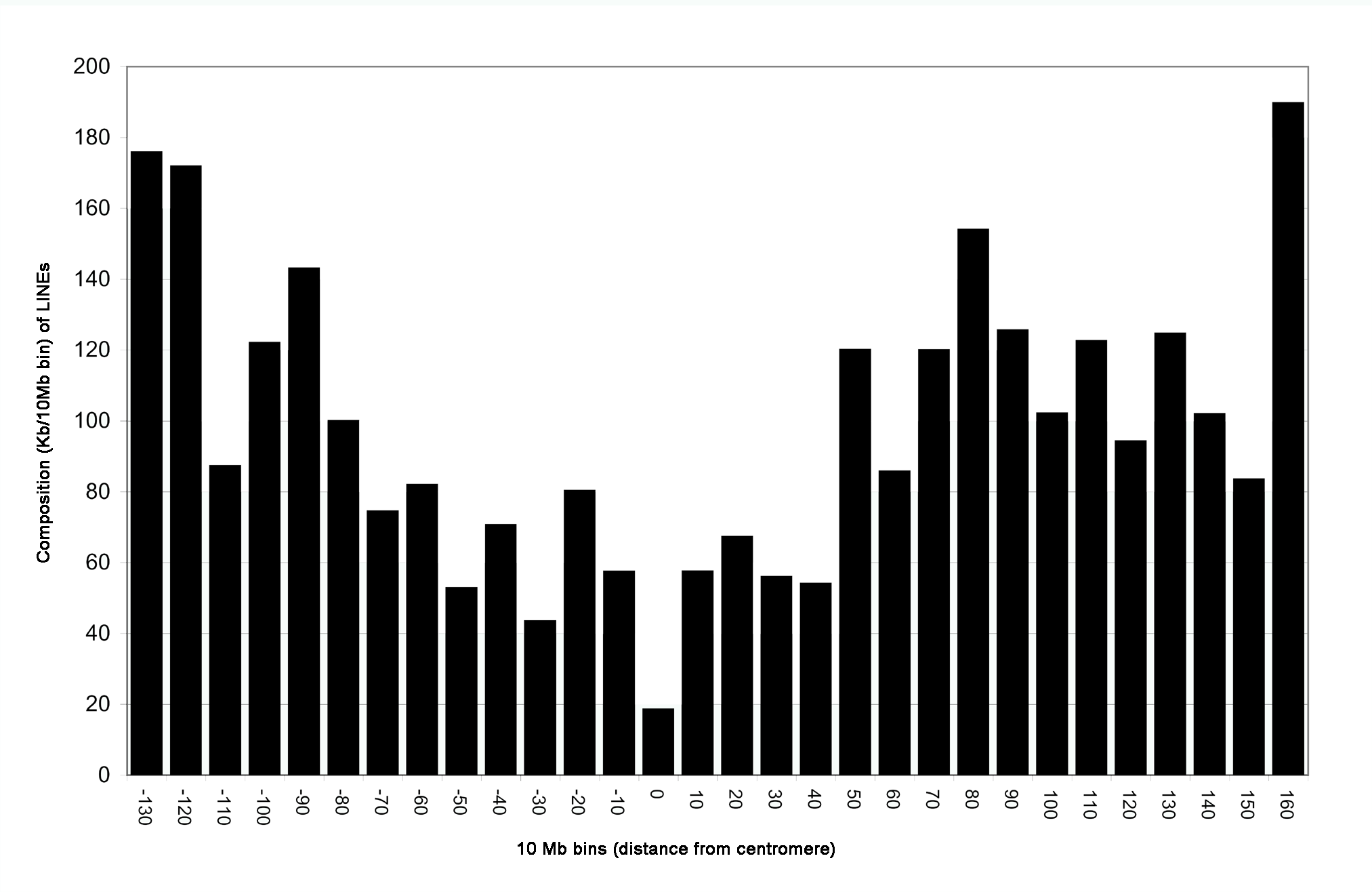

Supplement: Figure S3 — Distribution of LINEs across chromosome 1. (8.01 MB TIF) [file pgen.1000732.s003.tif]

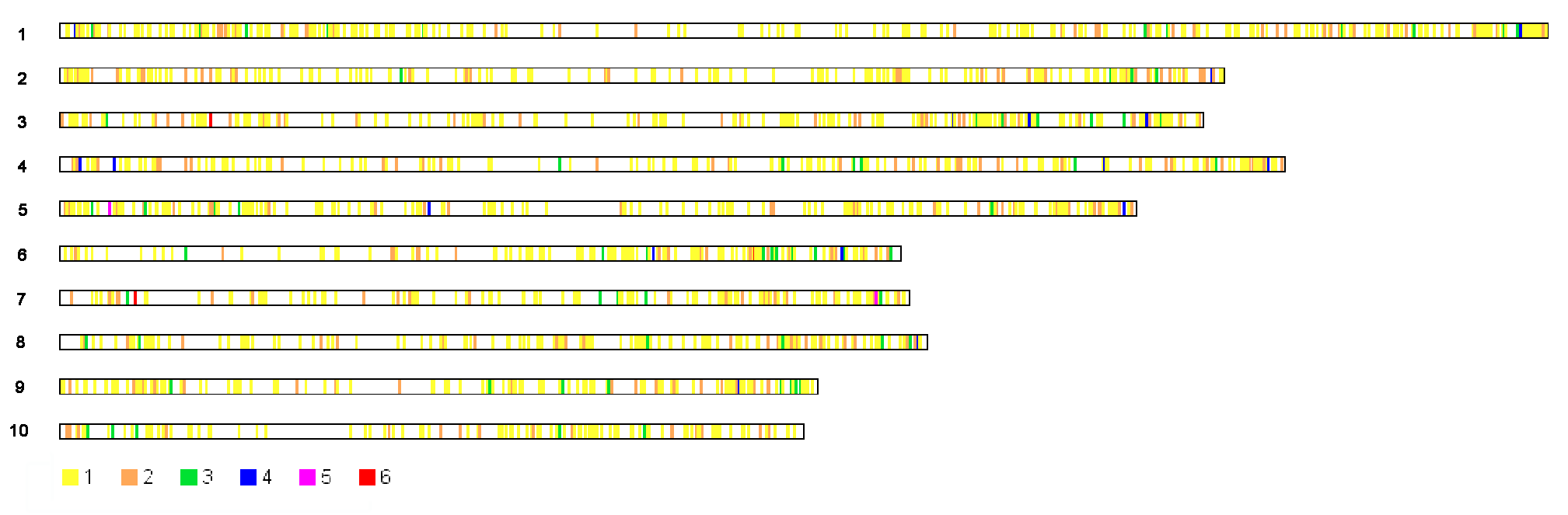

Supplement: Figure S4 — The general distribution of SINEs across the maize chromosomes. Different colors indicate different SINE families, as indicated in the figure. (0.16 MB TIF) [file pgen.1000732.s004.tif]

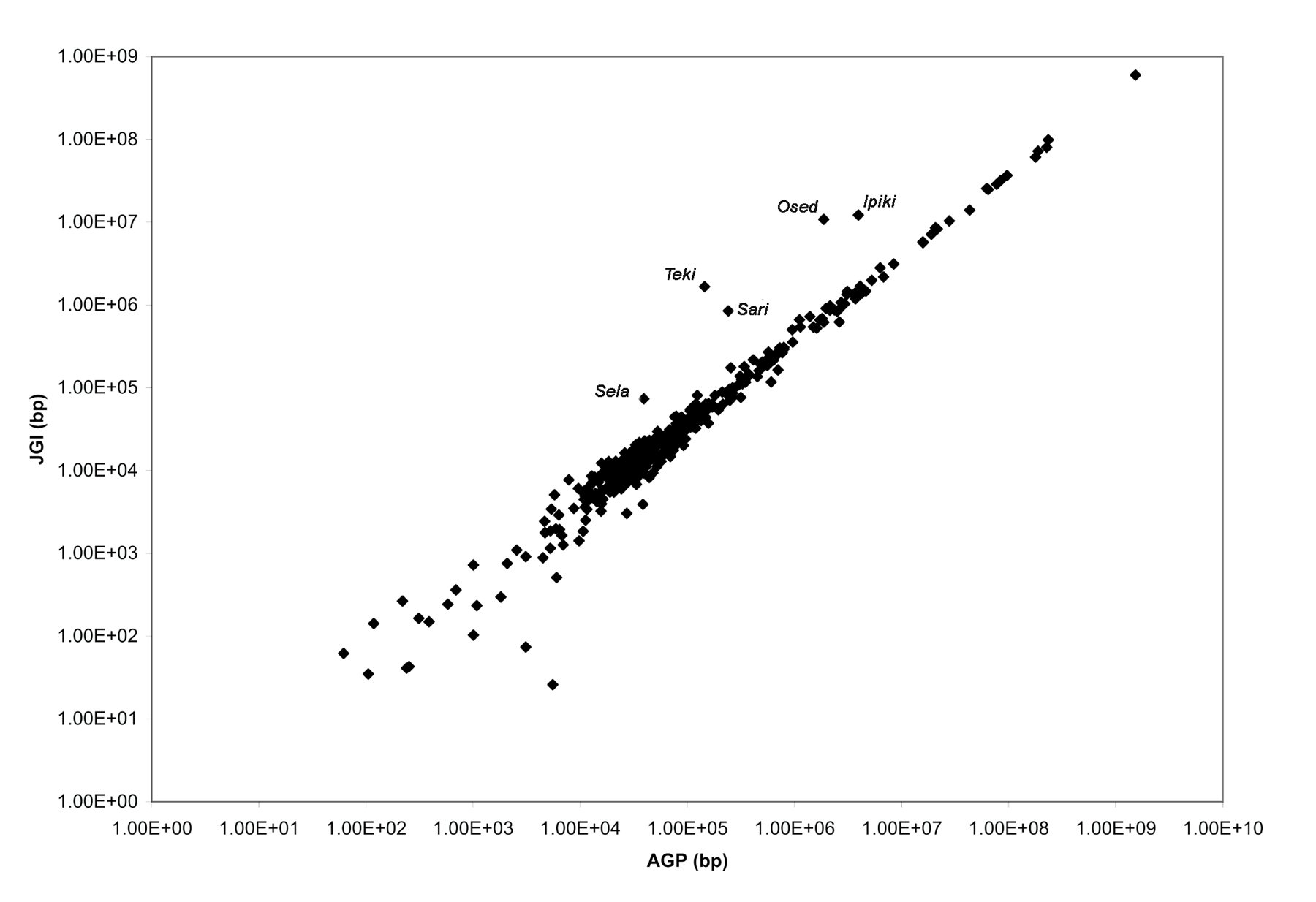

Supplement: Figure S5 — The relationship between the abundance of LTR retrotransposon families found within the AGP compared to their abundance in the sample sequence. Significant outliers are noted on the figure. (0.31 MB TIF) [file pgen.1000732.s005.tif]
